# Patterns of Childhood Abuse and Neglect in a Representative German Population Sample

**DOI:** 10.1371/journal.pone.0159510

**Published:** 2016-07-21

**Authors:** Christoph Schilling, Kerstin Weidner, Elmar Brähler, Heide Glaesmer, Winfried Häuser, Karin Pöhlmann

**Affiliations:** 1 Medizinische Fakultät Carl Gustav Carus, Technische Universität Dresden, Dresden, Germany; 2 Abteilung für Medizinische Psychologie und Medizinische Soziologie, Universität Leipzig, Leipzig, Germany; 3 Klinik und Poliklinik für Psychosomatische Medizin und Psychotherapie, Universitätsklinikum Mainz, Mainz, Germany; 4 Klinik für Innere Medizin I (Gastroenterologie, Hepatologie, Stoffwechsel- und Infektionskrankheiten, Psychosomatik, Klinikum Saarbrücken gGmbH, Saarbrücken, Germany; 5 Klinik für Psychosomatische Medizin und Psychotherapie, Technische Universität München, München, Germany; Central Institute of Mental Health, GERMANY

## Abstract

**Background:**

Different types of childhood maltreatment, like emotional abuse, emotional neglect, physical abuse, physical neglect and sexual abuse are interrelated because of their co-occurrence. Different patterns of childhood abuse and neglect are associated with the degree of severity of mental disorders in adulthood. The purpose of this study was (a) to identify different patterns of childhood maltreatment in a representative German community sample, (b) to replicate the patterns of childhood neglect and abuse recently found in a clinical German sample, (c) to examine whether participants reporting exposure to specific patterns of child maltreatment would report different levels of psychological distress, and (d) to compare the results of the typological approach and the results of a cumulative risk model based on our data set.

**Methods:**

In a cross-sectional survey conducted in 2010, a representative random sample of 2504 German participants aged between 14 and 92 years completed the Childhood Trauma Questionnaire (CTQ). General anxiety and depression were assessed by standardized questionnaires (GAD-2, PHQ-2). Cluster analysis was conducted with the CTQ-subscales to identify different patterns of childhood maltreatment.

**Results:**

Three different patterns of childhood abuse and neglect could be identified by cluster analysis. Cluster one showed low values on all CTQ-scales. Cluster two showed high values in emotional and physical neglect. Only cluster three showed high values in physical and sexual abuse. The three patterns of childhood maltreatment showed different degrees of depression (PHQ-2) and anxiety (GAD-2). Cluster one showed lowest levels of psychological distress, cluster three showed highest levels of mental distress.

**Conclusion:**

The results show that different types of childhood maltreatment are interrelated and can be grouped into specific patterns of childhood abuse and neglect, which are associated with differing severity of psychological distress in adulthood. The results correspond to those recently found in a German clinical sample and support a typological approach in the research of maltreatment. While cumulative risk models focus on the number of maltreatment types, the typological approach takes the number as well as the severity of the maltreatment types into account. Thus, specific patterns of maltreatment can be examined with regard to specific long-term psychological consequences.

## Background

Childhood maltreatment comprises acts of commission like emotional, physical and sexual abuse and acts of omission like emotional and physical neglect by a parent or a caregiver that results in harm, potential for harm, or threat of harm to a child [1].

Adverse childhood experiences are connected with the development of mental disorders such as schizophrenia [2], depression [3], substance related disorders [4], bipolar disorders [5] and post-traumatic stress disorders [6], as well as physical diseases like cardiovascular diseases [7], rheumatoid arthritis [8], autoimmune diseases [9] or cancer [10] in adulthood.

Several different approaches have been taken in the area of childhood maltreatment research. The first type of study examines the impact of a single type of childhood maltreatment on a distinct clinical outcome [11–14]. This approach implies that different types of childhood maltreatment occur separately and result in a specific psychological or physical outcome. This ignores findings that different types of child maltreatment co-occur rather than happen in isolation [15]. In community samples 13.5% to 43.3% [16–19] experienced multiple forms of maltreatment, whilst in clinical samples the rates of co-occurrence were even higher, namely 33%-95% [20, 21]. Furthermore these studies predominately examined the impact of acts of commission like sexual abuse and physical abuse [22, 23] and did not take into account the effect of acts of omission like emotional and physical neglect. In the last years, more attention was focused on the impact of non-sexual types of child maltreatment on the development of mental disorders [24–27]. There is strong evidence that emotional abuse and neglect also increase the occurrence of depressive disorders, anxiety disorders, eating disorders, drug use, alcohol use and suicidal behavior [28].

The second type of study examines the co-occurrence of different types of childhood maltreatment according to a cumulative risk model [29–34]. These studies concentrated on the cumulative effect of adverse childhood experiences. They found a strong graded relationship between the number of adverse experiences in childhood and the severity of mental disorder in adulthood [30, 32]. This approach implies that the extent of maltreatment is always the sum of the effects of the individual types of maltreatment, that each single type of maltreatment contributes equally to the level of general maltreatment, and that they are arbitrarily interchangeable. That approach seems to be static rather than interactive, since it ignores specific relations between particular types of maltreatment [35], for example the interaction between the number of maltreatment types and the severity of the maltreatment types [36]. There is evidence that specific combinations of particular types of childhood maltreatment have a stronger effect on clinical outcomes compared to other combinations [37–39].

A third type of study follows a typological approach by identifying specific patterns of single types of childhood maltreatment [35, 40–44]. This person-centered approach inductively examines the relations and interactions between different types of childhood maltreatment. Maltreatment is not the sum of the different single types of abuse and neglect, it is the pattern of the relations and interactions between them. Some studies following this approach used latent class models to empirically identify specific constellations of types of maltreatment [35, 40, 42, 43, 45], others used cluster analysis [41, 44, 46].

A recent German study of childhood abuse and neglect in a clinical sample based on cluster analysis identified three different patterns of childhood neglect and abuse [41]. The patterns were defined as a set of interrelated forms of childhood abuse or neglect including sexual abuse, physical abuse, physical neglect, emotional abuse and emotional neglect. The patterns were associated with varying degrees of psychiatric comorbidity, depression and general distress in adulthood. In pattern 1, participants that were neither abused in any form, nor neglected showed lowest values in the clinical outcomes. In pattern 2 participants that were emotionally abused and neglected but not physically or sexually abused showed more severe mental disorder. Pattern 3 comprised patients that were sexually and physically abused and also had experienced severe emotional abuse and neglect. They exhibited the highest severity of mental illness [41]. The results demonstrate that different types of childhood maltreatment are closely linked and tend to occur in specific constellations and not at random. The results also show that not only the cumulative effect of multiple adverse childhood experiences is related to the severity of mental disorder in adulthood, but also the specific composition of different types of childhood abuse and neglect. Thus far it remains unclear whether these patterns of childhood abuse and neglect are unique for the clinical sample or if they can be replicated in other samples.

The purpose of this study was (a) to identify different patterns of childhood maltreatment in a representative German community sample, (b) to replicate the patterns of childhood neglect and abuse recently found in a German clinical sample, (c) to examine whether participants reporting exposure to specific patterns of child maltreatment would report different levels of psychological distress, and (d) to compare the results of the typological approach and the results of a cumulative risk model based on our data set.

## Methods

### Sample Characteristics

Data was collected in a cross-sectional survey conducted by the independent institute for opinion and social research in Berlin, Germany (USUMA) in 2010. Participants were selected at random to generate a representative German sample set, with inclusion criteria being (a) age ≥ 14 years of age and (b) sufficient comprehension of the German written language. The survey was conducted through face to face interviews. All participants were informed about the design and the goals of the survey and gave informed written consent. For minor participants, written informed consent was also obtained from one parent. The study adhered to the ethical guidelines of the ICC/ESOMAR International Code of Marketing and Social Research Practice [47]. The survey was routinely approved by the institutional review board of the independent institute for opinion and social research in Berlin, Germany (USUMA). Quality and ethical compliance was ensured through qualified personnel, experienced interviewers, and experts in the fields of sociology, psychology, economics, mathematics and computer science. In the survey no identifiable information such as addresses, emails or names were collected.

The present study posed a low risk to the participants. An additional ethical approval was not required as procedures such as medical treatments, invasive diagnostics or those causing psychological, spiritual or social harm or discomfort for the participants were not involved.

The initial sample set comprised 4455 participants aged between 14 and 92 years. 15.6% of the target population declined to participate in the survey, 28.4% could not be reached. The response rate was 56%, i.e. 2504 participants finally completed the survey. A total of 2500 participants completed the Childhood Trauma Questionnaire. The sample characteristics are summarized in Table 1. For further details of the sample characteristics and the data assessment see Häuser et al. 2011 [18].

**Table 1:** Characteristics of the 3 clustersolution.

		**Total sample**	**Cluster 1**	**Cluster 2**	**Cluster 3**	**F / Χ²**	**p**	**effect-size**	**Post-hoc (Bonferroni)**
N		2493	1761	628	104				
			70.6%	25.2%	4.2%				
age (years) 14–92	M	50.6	49.5	53.5	52.9	F = 11.960	< .001	ɳ^2^ = .010	c1<c2*** / c2>c3 n.s. / c1<c3 n.s.
	SD	18.6	18.7	17.7	18.8				
Sex	male	1169 (46.9%)	819	305	45	Χ² = 1.360	.507		
	female	1324 (53.1%)	942	323	59				
emotionalabuse	M	6.51	5.51	7.93	14.80	F = 1906.382	< .001	ɳ^2^ = .605	c1<c2*** / c2<c3*** / c1<c3***
	SD	2.60	.92	2.46	3.61				
Physical abuse	M	5.88	5.37	6.54	13.62	F = 2100.728	< .001	ɳ^2^ = .926	c1<c2*** / c2<c3*** / c1<c3***
	SD	2.17	.65	1.81	3.91				
sexual abuse	M	5.45	5.04	5.79	10.22	F = 846.560	< .001	ɳ^2^ = .405	c1<c2*** / c2<c3*** / c1<c3***
	SD	1.65	.26	1.57	4.77				
emotionalneglect	M	10.09	8.27	14.03	17.07	F = 1072.039	< .001	ɳ^2^ = .463	c1<c2*** / c2<c3*** / c1<c3***
	SD	4.24	.26	4.18	3.88				
physical neglect	M	8.14	6.99	10.31	14.55	F = 906.636	< .001	ɳ^2^ = .421	c1<c2*** / c2<c3*** / c1<c3***
	SD	3.02	2.02	2.78	3.32				
		**Total sample**	**Cluster 1**	**Cluster 2**	**Cluster 3**	**F**	**p**	**effect-size**	**Contrasts (simple)**
Depression PHQ-2	M	0.86	0.62	1.34	2.03	173.180	< .001	ɳ^2^ = .122	c1<c2*** / c2<c3*** / c1<c3***
	SD	1.13	0.96	1.28	1.23				
Anxiety GAD-2	M	0.67	0.44	1.08	2.16	209.256	< .001	ɳ^2^ = .144	c1<c2*** / c2<c3*** / c1<c3***
	SD	1.10	0.84	1.31	1.53				
psych. Distress total score	M	1.53	1.05	2.41	4.19	225.274	< .001	ɳ^2^ = .154	c1<c2*** / c2<c3*** / c1<c3***
	SD	2.06	1,64	2.41	2.51				

***p < .001.

### Assessment

#### Childhood trauma questionnaire [48]

The Childhood Trauma Questionnaire is a 28-item self-report inventory that provides a brief, reliable, and valid screening for childhood abuse and neglect. It assesses five types of traumatization—emotional, physical, and sexual abuse, and emotional and physical neglect. Each scale contains 5 items. A five point frequency of occurrence scale is utilized: (1) never true, (2) rarely true, (3) sometimes true, (4) often true, and (5) very often true. The following cut-off values for traumatization were suggested by the authors: emotional abuse 13, emotional neglect 15, physical abuse 10, physical neglect 10, and sexual abuse 8.

The validated German short form of the CTQ [49] was used. Four scales show good internal consistency (emotional abuse α = .87, physical abuse α = .80, emotional neglect α = .83, sexual abuse α = .83), one scale lacks internal consistency (physical neglect α = .55).

#### Patient health questionnaire [50]

The Patient Health Questionnaire-4 is an ultra-short screening tool for depression and anxiety. The PHQ-4 combines two validated two item screeners. Two items assess depression (PHQ-2), two items assess general anxiety (GAD-2). For the overall psychological distress a PHQ-4 total score can be calculated. Answer categories are (0) not at all, (1) several days, (2) more than half the days, and (3) nearly every day. A score ≥3 indicates a higher risk for a mental illness (“yellow flag”). A GAD-2 score ≥3 has a sensitivity of 86% and a specificity of 83% for generalised anxiety disorder, a sensitivity of 76% and a specificity of 81% for panic disorder, a sensitivity of 70% and a specificity of 81% for social anxiety disorder, a sensitivity of 59% and a specificity of 81% for posttraumatic stress disorder, and a sensitivity of 65% and a specificity of 88% for any anxiety disorder. [51, 52]. The validated German version [51] was used.

### Statistical Analyses

A cluster analysis was conducted to identify patterns of childhood traumatization. A two-step cluster analysis was chosen because of the large sample size. Based on the log-likelihood distance measure and the Bayesian Information Criterion (BIC) the number of clusters was determined.

The silhouette coefficient was used to quantify the goodness of fit of the cluster solution. This coefficient is a measure for both the cohesion within a cluster, and separation between clusters. For each element in a cluster, the average distance to all other elements in its cluster and the average distance to all elements in each of the other clusters are calculated. For each element, the silhouette measure is the difference between the smallest average between cluster distance and the average within cluster distance, divided by the larger of the two distances. In a good solution, the within cluster distances are small and the between cluster distances are large, resulting in a silhouette measure close to maximum of 1. The silhouette coefficient ranges from -1 to +1. Results can be classified as “good” (>.5), “fair” (.2 <>.5) or “poor” (< .2) [53].

General linear model (GLM) was used to examine differences in depression, anxiety and overall psychological distress between the patterns. Contrasts and post-hoc tests were performed to identify differences between the single clusters. For some variables the assumptions for variance analysis (normal distribution, variance homogeneity) were not fulfilled. Variance analysis is considered a robust test against the normality assumption. This means that it tolerates violations to its normality assumption rather well. Small differences easily reach statistical significance particularly in large samples. The general linear model with means and standard deviations was used for normally distributed variables, with the chi square test used for categorical variables. Standardized residuals are reported to identify differences between the different patterns. Positive residuals indicate over-representation, negative residuals indicate under-representation.

Data was analysed with the Software Package for Social Sciences for Windows (SPSS), version 21.

## Results

### Cluster-Analysis

The cluster-analysis led to a three cluster solution. Cluster 1 comprised 1761 participants (70.6%), cluster 2 628 (25.2%) and cluster 3 104 (4.2%). The goodness of fit of the three cluster solution can be considered between good and fair (silhouette coefficient = .5). To ensure that the three cluster solution was the most appropriate cluster solution, the silhouette coefficients for a two and a four cluster solution were calculated. The silhouette coefficient for the two cluster solution was .5, and for the four cluster solution .4. Since the goodness of fit of the three cluster solutions was comparable, content related criteria had to be used to determine, which cluster solution was the most appropriate. The two cluster solution would be too reductionist, since it ignores the difference between different patterns of abuse and neglect. The four cluster solution would be too complex and difficult to differentiate. Therefore the three cluster solution is considered the most appropriate cluster solution.

Differences between the clusters in the sociodemographic variables for age and sex were tested.

There were no differences in sex (χ ² = 1.360, p = .507).

Differences were found for age (F = 11.960, p <0.001). There were highly significant differences in age between cluster 1 and cluster 2, indicating lower age for cluster 1. No differences in age were found between cluster 2 and cluster 3 or between cluster 1 and cluster 3.

In cluster 1 all CTQ scales had very low values. Only emotional neglect and physical neglect scores were slightly increased. All scales were below the cut-off values provided by the authors of the CTQ [48]. In cluster 2, all scales reached values higher than the means of the scales in cluster 1. Nevertheless, emotional abuse, physical abuse and sexual abuse showed relatively low values. The scores were below the cut-off values of the CTQ. Emotional neglect and physical neglect were increased in cluster 2. Physical neglect was above the CTQ cut-off value, emotional neglect was slightly below the cut-off score. In cluster 3, all CTQ scales showed high values. All scales were above the cut-off values. While the scales of abuse (emotional, physical and sexual) were relatively low in cluster 1 and cluster 2, they were markedly increased in cluster 3. In the post hoc analysis highly significant differences were found between the three clusters for all CTQ scales, indicating lowest values in cluster 1 and highest values in cluster 3.

Characteristics of the three clusters are summarized in Table 1.

### Differences in depression and anxiety between the clusters

Highly significant differences for depression (PHQ-2) could be found between the three clusters (F = 173.180, p < .001). Participants in cluster 1 were hardly depressed, participants in cluster 2 were significantly more depressed, participants in cluster 3 showed the highest degree of depression.

Highly significant differences could be found for anxiety (GAD-2) between the three clusters (F = 209.1256, p < .001). Participants in cluster 1 showed the least degree of anxiety, participants in cluster 2 were significantly more anxious, participants in cluster 3 showed the highest degree of anxiety.

Highly significant differences could be found for overall mental distress (PHQ-4) between the three clusters (F = 173.180, p < .001). Participants in cluster 1 showed the lowest degree of overall psychological distress, participants in clusters 3 had the highest degree of overall psychological distress.

Results are summarized in Table 1

Cases that scored > = 3 in the two PHQ subscales PHQ-2 and GAD-2 were labeled as “yellow flag”. Cases > = 6 in general psychological distress (PHQ-4) were also labeled as “yellow flag”. A yellow flag indicates a higher risk for mental illness and is an indicator that additional diagnostic information is needed and appropriate treatment should be considered.

Highly significant differences could be found between the three clusters with regard to the proportion of cases that scored > = 3 in the PHQ-2 and the GAD-2. Individual comparisons of the three groups (cluster 1- cluster 2, cluster 2- cluster 3, and cluster 1- cluster 3) also revealed highly significant differences.

In the PHQ-2 screening for depression, 3.9% in cluster 1, 14.5% in cluster 2 and 32.7% in cluster 3 reached yellow flag scores. In the GAD-2 screening, 2.8% in cluster 1, 13.3% in cluster 2 and 43.2% in cluster 3 reached yellow flag scores.

Highly significant differences could be found between the three clusters with regard to the proportion of cases that scored > = 6 in general psychological distress (PHQ-4). Individual comparisons of the three groups (cluster 1- cluster 2, cluster 2- cluster 3, and cluster 1- cluster 3) revealed highly significant differences. In the PHQ-4 screening for general psychological distress, 2.3% in cluster 1, 11.3% in cluster 2 and 31.7% in cluster 3 reached yellow flag scores.

There were large effect sizes for the differences between the three CTQ cluster for depression, generalized anxiety and general psychological distress.

Results are summarized in Table 2.

**Table 2:** Distribution of yellow flags in PHQ-2 and GAD-2 in the three CTQ-clusters.

		Total sample	cluster 1	cluster 2	cluster 3	*χ*²	partial eta^2^	standardized residuals
PHQ-2> = 3		193	68	91	34	167.615***	.256	C1 = -5.9 / C2 = 6.1 /C3 = 9.1
	%	7.8	3.9	14.5	32.7			
C1 –C2						84.330***	.188	
C2 –C3						20.715***	.168	
C1 –C3						157.448***	.291	
GAD-2 > = 3		177	49	83	45	290.788***	.321	C1 = -6.8 / C2 = 5.8 / C3 = 13.8
	%	7.1	2.8	13.3	43.3			
C1 –C2						96.532***	.201	
C2 –C3						55.548***	.276	
C1 –C3						335.072***	.425	
PHQ-4> = 6		144	40	71	33	203.749***	.276	C1 = -6.1 / C2 = 5.8 / C3 = 11
	%	5.8	2.3	11.3	31.7			
C1—C2						85.280***	1.89	
C2—C3						30.538***	2.04	
C1—C3						226.598***	3.49	

***p < .001.

### Comparison of the typological approach and the cumulative risk model

To compare the typological approach and the cumulative risk model based on our data we applied a cumulative risk model to our data. For each participant in the sample each type of maltreatment was coded with 0 if the participant was below the cut-off of the CTQ scale and 1 if it reached the cut-off. The adverse child experience (ACE) score was then calculated by the sum of the five subscales. The ACE-Score ranged from 0 (no type of maltreatment) to 5 (all types of maltreatment). Detailed information about the number of reported types of maltreatment in the three CTQ Clusters are summarized in Table 3.

**Table 3:** Number of reported types of maltreatment in the three CTQ clusters.

Number of reported types of maltreatment		Total sample	Cluster1	Cluster 2	Cluster 3
0	Number	1616	1505	111	0
	%	64.8	85.5	17.7	0
1	Number	535	256	278	1
	%	21.5	14.5	44.3	1
2	Number	204	0	31.5	6
	%	8.2	0	198	5.8
3	Number	54	0	37	17
	%	2.2	0	5.9	16.3
4	Number	58	0	4	54
	%	2.3	0	.6	51.9
5	Number	26	0	0	26
	%	1.0	0	0	25

To compare the goodness of the typological approach and the cumulative risk model based on our data set, we compared the explained variance of the dependent variables (PHQ-4, PHQ-2, GAD-2) in both models. Therefore we conducted a linear regression analysis with the ACE-scores as independent variables and the PHQ-4, PHQ-2 and GAD-2 as dependent variables. The ACE-sores explained 14.5% of the variance of the PHQ-4 (R^2^ = .145), 10.8% of PHQ-2 (R^2^ = .108) and 14.3% of GAD-2 (R^2^ = .143). In the typological model the three maltreatment clusters explained slightly more variance of the dependent variables. The clusters explained 15.4% of the variance of the PHQ-4 (R^2^ = .154), 12.2% of PHQ-2 (R^2^ = .122) and 14.4% of GAD-2 (R^2^ = .144). Although the two approaches apply different strategies to assess differences in maltreatment (ACE-score & cut-off values versus inductively identified patterns) they can explain nearly the same proportion of variance of psychological distress.

## Discussion

The purpose of this study was (a) to identify different patterns of childhood maltreatment in a representative German community sample, (b) to replicate the patterns of childhood neglect and abuse recently found in a German clinical sample, (c) to examine whether participants reporting exposure to specific patterns of child maltreatment would report different levels of psychological distress, and (d) to compare the results of the typological approach and the results of a cumulative risk model based on our data set.

### Patterns of childhood abuse and neglect in the community sample

Three qualitatively different patterns of childhood abuse and neglect were identified. The first group comprised participants reporting low levels of abuse and neglect in childhood. The second pattern included participants that reported neglect both emotional and physical but not abuse (emotional, physical or sexual). The third pattern comprised participants that reported neglect as well as abuse in childhood.

### Correspondence to the patterns of a clinical sample

There were strong similarities between the three patterns of childhood abuse and neglect that were recently reported for a large German clinical sample [41]. Similar to cluster 1 in the clinical sample, cluster 1 in the representative sample comprised participants with mild forms of neglect or abuse with the exception of physical neglect, which was increased in all three clusters in the clinical sample. Cluster 3 of the two samples also corresponded. In both samples cluster 3 showed values above the cut-off defined for moderate to high severity for all types of neglect and abuse. Cluster 2 also showed clear parallels in the two samples. Similar to cluster 2 in the clinical sample, cluster 2 in the representative sample showed no severe physical or sexual abuse. In both samples physical neglect was moderate to severe in cluster 2. In summary, the comparison of the cluster solutions of the two different samples showed strong similarities. In particular cluster 1 and cluster 3 had a high degree of correspondence between the two samples.

In a few areas, the patterns of the community sample diverged from the patterns of the clinical sample. There was a difference in emotional abuse in cluster 2 between the two samples.

In the representative sample in cluster 2 only emotional and physical neglect were increased, whereas cluster 2 in the clinical sample showed high values for emotional and physical neglect and emotional abuse.

A second difference concerned the extent of physical neglect. In the representative sample there were differences in physical neglect between the three patterns. Cluster 1 showed the lowest values in physical neglect, cluster 3 had the highest values. In the clinical sample, the three patterns could not be differentiated by physical neglect as values for physical neglect were increased equally in all three patterns. There are two explanations for the two differences: Firstly, there might be a psychometric artefact because of the weak internal consistency of the CTQ scale physical neglect [54]. Neglect types are usually much harder to define than abuse types so the assessment of neglect often lacks internal consistency [55]. One consequence for our results could be that the physical neglect type does not sufficiently differentiate the cases into different patterns of abuse and neglect. Secondly, the differences in physical neglect and emotional abuse might reflect the differences of sample characteristics. In the community sample, 7–8% met the criteria for a yellow flag for anxiety and depression in the PHQ-4. In contrast, in the clinical sample, 58% met the criteria for a probable depressive disorder and 42% for a probable anxiety disorder. This might indicate that the clinical sample was a highly selected sample with participants that had a higher degree of depression, anxiety and psychological distress and also reported more severe childhood maltreatment like emotional abuse. The comparison between the two samples showed higher scores for all CTQ scales in the clinical sample. That was particularly the case for emotional abuse, which was almost twice as high in the clinical sample (M = 11.86; SD = 5.9 and M = 6.51; SD = 2.6, respectively, p < .001; d = 1.47).

There were also differences in the distribution of the three clusters between the two samples. In the community sample more than 70% of the cases were allocated to cluster 1, 25% to cluster 2 and 4% to cluster 3. In the clinical sample 44% of the cases were allocated to cluster 1, 32% to cluster 2 and 23% to cluster 3. That might illustrate that the community sample was a representative sample and that the clinical sample comprised participants typical for the secondary care setting.

In summary, there was a high degree of correspondence between the patterns of different types of childhood abuse and neglect identified in this representative sample and the ones described in a large German clinical sample.

The results might provide an indication that very often childhood maltreatment is not the result of one single type of maltreatment. In the representative sample, approximately 30% (cluster 2 and cluster 3) experienced more than one type of maltreatment, in the clinical sample that rate was 55%. The co-occurrence of different subtypes of maltreatment is consistent with the findings of numerous studies [15, 21, 34].

Furthermore the results are in line with the findings of other studies that identified patterns of childhood maltreatment by conducting cluster-analysis or latent class models. Most of them also identified a pattern with low maltreatment with low values for all applied types of maltreatment and a pattern with high maltreatment with high values for all applied types of maltreatment [40, 42, 56].

A study measuring sexual abuse, physical punishment and parental neglect reported a three cluster solution with low maltreatment, intermediate maltreatment and high maltreatment [56]. The cluster with low maltreatment showed low levels of maltreatment in all three maltreatment types, the cluster with intermediate maltreatment showed moderate levels of maltreatment for all three scales and the cluster with high maltreatment showed high levels of maltreatment for all three maltreatment types. In particular, sexual abuse was high in the high maltreatment group. Another study reanalysed three former studies that assessed multi-maltreatment by using a typological approach [44]. The number of the five maltreatment types (sexual abuse, physical abuse, psychological maltreatment, neglect and witnessing family violence) and the degree of maltreatment (frequency, duration and severity) were assessed. Cluster analysis revealed three clusters of maltreatment types: low, moderate, and high maltreatment. The authors found that maltreatment is best grouped according to the degree of maltreatment and not by the number of maltreatment types. A Californian study with high-risk adolescents assessed physical, sexual and emotional maltreatment using latent profile analysis found three maltreatment classes [40]. The first class comprised cases with high values in all three maltreatment types, the second class had high levels of physical and emotional maltreatment, and the third class had low levels in all three types. A further study examined physical abuse, sexual abuse, emotional abuse and domestic violence exposure [35]. A stepwise latent class analysis was conducted. In the first step, a two class solution divided the sample into two groups—maltreatment versus no maltreatment. In the second step, within the maltreatment group four classes were identified (sexual abuse, physical abuse, emotional abuse, domestic violence exposure). In the third step four multi-maltreatment classes were identified (violent home, hostile home, harsh parenting, and sexual abuse).

The comparability of the studies is restricted because of sample differences and differences in the number and type of childhood maltreatment used in the different studies. Similar to our three cluster solution numerous studies using the typological approach identify three patterns of maltreatment. Sexual abuse seems to be predominant in the most severe maltreatment group. The finding that the more severe types of maltreatment, like sexual abuse, are accompanied by a higher severity of the other types of maltreatment, like emotional neglect, might indicate a relationship between the number of types of maltreatment and the severity of these types [36]. This combination of number of types of maltreatment and the severity of these types may be the link between the findings of the cumulative risk model and the typological approach.

The results of our three cluster solution partly correspond with those results of the cumulative risk model, that indicate that there is a strong graded relationship between the number of types of maltreatment and the severity of psychological distress. In the typological approach, in contrast to the cumulative risk model, the single types of maltreatment are not interchangeable because they are specifically interrelated. While neglect can occur without physical or sexual abuse, it seems to be unlikely that physical and sexual abuse occur independent of neglect.

### Comparison of the typological approach with the cumulative risk model

The comparison of the results of the typological approach and the cumulative risk model showed that in the typological model the three maltreatment clusters explained slightly more variance of psychological distress and, in some instances, the two models partly deliver different predictions of the severity of maltreatment. While participant A with emotional abuse and emotional neglect and a participant B with sexual abuse and emotional neglect would get the same ACE score (2) in the cumulative risk model, participant A would be allocated to cluster 2 and the participant B to cluster 3 in the typological approach. While participant B would get a relatively low ACE score in the cumulative risk model (2/5), the same participant would be allocated to the most severe cluster in the typological approach.

One explanation for the different predictions might be that the cumulative risk model calculates the number of existing or non-existent types of maltreatment, while the typological approach also covers the severity of all the measured types of maltreatment. Because dichotomous variables are used in the cumulative risk model, there is no metric information about the severity of the single types of maltreatment. Furthermore the dichotomization of metric data normally needs a cut-off and there might be some degree of uncertainty in determining a cut-off. A cut-off might be a consensual agreement of a research community or it might be determined by empirical or theoretical conclusions. Therefore the value from which a participant has to be considered maltreated is relative. The advantage of the typological approach is that instead of using cut-off values, empirical patterns of childhood are identified inductively. A further divergence of the two approaches addresses the question of how the single types contribute to maltreatment as a whole. While the cumulative risk model implies that all single types contribute equally to maltreatment, the results of this study using the typological approach indicate that especially physical and sexual abuse, in particular, are more severe types of maltreatment than types of neglect.

The results support a typological approach that goes beyond a mere cumulative risk model. Inductively identified patterns of childhood maltreatment facilitate formulating hypotheses for the co-occurrence of single types of maltreatment. We hypothesize that the patterns of maltreatment might reflect specific constellations of environmental conditions of the maltreated child, especially of the relationship to the caregiver. For example cluster 2 comprised participants who experienced several forms of neglect simultaneously. That might reflect a relationship to a neglectful but non-abusing caregiver, for example a depressive mother that lacks perception of the emotional or physical needs of the child. Cluster 3 indicated that sexual and physical abuse seem to be systematically accompanied by severe forms of emotional and physical abuse and neglect. That might illustrate that it is rather common that sexual or physical abuse are associated with emotionally and physically neglectful behaviour of the caregiver.

### Severity of depression and anxiety within the patterns

The three patterns showed highly significant differences in the severity of depression and general anxiety. Participants reporting low levels of abuse and neglect in childhood (cluster 1) showed lowest severity of the clinical outcomes, participants that reported neglect showed moderate clinical outcomes and participants that reported neglect and abuse (cluster 3) showed highest severity. Even though the PHQ is a screening instrument and cannot provide standardized diagnoses, it shows a high sensitivity and specificity in predicting standardized depression diagnosis [57]. These results are in line with previous findings on the impact of multiple childhood traumatizations on the severity of clinical outcomes [41, 58–60]. The result, that not only physical or sexual abuse but also forms of neglect have an impact on the severity of mental disorder in adulthood, is in accordance with the findings from recent studies that examined the impact of non-sexual forms of maltreatment on later clinical outcomes [24–26, 28].

The results are partly consistent with a cumulative risk model, as there is a continuous dose-response relationship, since low levels of abuse and neglect are related to low levels of psychological distress, only neglect to medium levels, and neglect and abuse to high levels of psychological distress.

The results are also in line with numerous studies applying the typological approach, which found differences in psychological outcomes between three different classes of maltreatment [35, 40, 44, 56]. One study found differences in symptoms of anxiety, depression, drug use and alcohol use between the three clusters [56]. In a further study that revealed three clusters the degree of maltreatment corresponded with the level of psychological maladjustment [44]. Another study found that psychological variables (withdrawal, somatic complaints, anxiety, depression, social problems, thought problems, attention problems, delinquent behaviour and aggressive behaviour) were significantly higher in the first class with high maltreatment [40]. Differences between class two and class three did not differ significantly. In another study emotional abuse was associated with general psychopathology (depression and anxiety), the combination of emotional abuse with physical abuse was associated with conduct-related symptoms (substance use, risky sexual behaviour) [35].

In the referenced studies, it was found that sexual abuse was usually predominant in the most severe clusters. That result stands in line with our results and with the recent finding, that the psychological consequences of child sexual abuse are more severe than those of non-sexual types of maltreatment [61].

In addition to the assumptions on the antecedents of child maltreatment the typological approach might facilitate formulating hypotheses for specific consequences of certain patterns of childhood abuse and neglect [62]. Similar to our findings, other studies showed that some combinations of types of maltreatment have worse consequences than others [21]. While our study could show quantitative differences in psychological distress for the three patterns of maltreatment, other studies could show differentiated results for specific combinations of maltreatment. One study found that a combination of physical and emotional abuse was associated with conduct-related problems (e.g. substance use, risky sexual behaviour), emotional abuse alone or in combination with other maltreatment types was especially associated with general psychopathology (e.g. anxiety, depression) [35]. Another study found that the combination of physical and sexual abuse was associated with a greater risk for antisocial an suicidal behaviour [37].

## Strengths and Limitations

A key strength of this study is that the three CTQ cluster solution is based on a very large representative community sample and that the results replicate recent findings of a highly similar cluster analytic differentiation in a large clinical sample [41]. Both samples cover a wide range of types and degrees of childhood traumatization since they are representative or include a wide range of mental disorders and comorbidities, respectively.

The accuracy of retrospectively self-reported childhood trauma might be limited by errors in recall because of possible inaccessibility of traumatic life events [63–65] or intentionally false positive or negative responding. However, there is also solid support for the reliability of the retrospective assessment of adverse childhood experiences [66–69].

Furthermore it is important to note that the low internal consistency of the physical neglect scale can affect the results. In the research of child maltreatment the definition of neglect types is usually harder than the definition of abuse types. While abuse types can be defined by specific behavior, neglect has to be defined by acts of omission [55].

The generalization of the results might be limited, since the response rate was only 56%. It is possible that participants refusing participation had relevant tendencies in childhood traumatization.

The PHQ-4 is only a short screening instrument for depression and general anxiety and does not diagnose a mental disorder according to DSM or ICD classification [50].

The survey was cross-sectional. Thus, developmental-psychological pathways from childhood maltreatment to the severity of mental symptoms in adulthood cannot be examined.

## Future Research Directions

There are several methodical implications for further studies examining the impact of maltreatment on clinical outcomes. Since single subtypes usually co-occur, studies should assess a wide range of maltreatment types. The different maltreatment types should be clearly defined, so that the shared variance of two types can be interpreted as their interrelatedness and not as a conceptual overlap of two insufficiently defined maltreatment types. This applies in particular for the different neglect types, since they are harder to define than abuse types. Studies comparing the cumulative risk model and the typological approach with the same data set are needed to explain the convergences and divergences of the two competitive models. Studies should especially examine if there is a reliable relationship between the number and the severity of the single maltreatment types. To date, there are only a few studies that focus on that interaction. Furthermore, studies should examine if specific patterns of maltreatment are reliably connected with specific antecedent environmental constellations and specific clinical outcomes.

## Conclusions

The results show that different types of childhood maltreatment are interrelated and can be grouped into specific patterns of childhood abuse and neglect, which are associated with differing severity of psychological distress in adulthood. The results correspond to those recently found in a German clinical sample and support a typological approach in the research of maltreatment. While cumulative risk models focus on the number of maltreatment types, the typological approach takes the number as well as the severity of the maltreatment types into account. Thus, specific patterns of maltreatment can be examined with regard to specific long-term psychological consequences.
